# Using Microphysiological System for the Development of Treatments for Joint Inflammation and Associated Cartilage Loss—A Pilot Study

**DOI:** 10.3390/biom13020384

**Published:** 2023-02-17

**Authors:** Meagan J. Makarczyk, Sophie Hines, Haruyo Yagi, Zhong Alan Li, Alyssa M. Aguglia, Justin Zbikowski, Anne-Marie Padget, Qi Gao, Bruce A. Bunnell, Stuart B. Goodman, Hang Lin

**Affiliations:** 1 Department of Orthopaedic Surgery, University of Pittsburgh School of Medicine, 450 Technology Drive, Rm 217, Pittsburgh, PA 15219, USA; 2 Department of Bioengineering, University of Pittsburgh Swanson School of Engineering, 450 Technology Drive, Rm 217, Pittsburgh, PA 15219, USA; 3 Department of Orthopaedic Surgery, Stanford University, Stanford, CA 94350, USA; 4 Department of Microbiology, Immunology, and Genetics University of North Texas Health Science Center, Fort Worth, TX 76107, USA

**Keywords:** osteoarthritis, tissue chip, microphysiological system, inflammation, DMOADs, BMP-7

## Abstract

Osteoarthritis (OA) is a painful and disabling joint disease affecting millions worldwide. The lack of clinically relevant models limits our ability to predict therapeutic outcomes prior to clinical trials, where most drugs fail. Therefore, there is a need for a model that accurately recapitulates the whole-joint disease nature of OA in humans. Emerging microphysiological systems provide a new opportunity. We recently established a miniature knee joint system, known as the miniJoint, in which human bone-marrow-derived mesenchymal stem cells (hBMSCs) were used to create an osteochondral complex, synovial-like fibrous tissue, and adipose tissue analogs. In this study, we explored the potential of the miniJoint in developing novel treatments for OA by testing the hypothesis that co-treatment with anti-inflammation and chondroinducing agents can suppress joint inflammation and associated cartilage degradation. Specifically, we created a “synovitis”-relevant OA model in the miniJoint by treating synovial-like tissues with interleukin-1β (IL-1β), and then a combined treatment of oligodeoxynucleotides (ODNs) suppressing the nuclear factor kappa beta (NF-κB) genetic pathway and bone morphogenic protein-7 (BMP-7) was introduced. The combined treatment with BMP-7 and ODNs reduced inflammation in the synovial-like fibrous tissue and showed an increase in glycosaminoglycan formation in the cartilage portion of the osteochondral complex. For the first time, this study demonstrated the potential of the miniJoint in developing disease-modifying OA drugs. The therapeutic efficacy of co-treatment with NF-κB ODNs and BMP-7 can be further validated in future clinical studies.

## 1. Introduction

Osteoarthritis (OA) is a highly prevalent joint disorder affecting millions of people worldwide [1] and is the 11th global contributor to disability [2]. Current treatment options for OA are primarily palliative methods, such as non-steroidal anti-inflammatory drugs (NSAIDs) for pain management [3]. No disease-modifying OA drugs (DMOADs) have reached FDA approval, limiting treatment options to surgical intervention, which is not suitable for all patients [4,5]. The unmet clinical need for effective DMOADs is primarily due to the lack of models to faithfully recapitulate the whole-joint disease nature of OA [6]. Methods such as two-dimensional cell cultures and human explants allow for high-throughput modeling but are limited in their viability and ability to replicate multi-tissue interactions. Animal models have been the most useful source of information for elucidating disease onset, the whole-joint pathology of OA, and testing therapeutics [6]. While necessary for understanding the complex pathological nature of OA, animal models are genetically and anatomically different from humans, limiting our ability to predict therapeutic outcomes [4,5,6]. A model that accurately predicts human responses to DMOADs before clinical trials could save significant costs and provide critical knowledge in the therapeutic development pipeline [4].

In the last decade, the idea of organs-on-chips, also known as microphysiological systems (MPSs), has become more prevalent in the tissue engineering realm [7,8]. These systems incorporate multiple tissue components of a specific organ system in vitro for assessing environmental stimuli and genetics on the tissues that constitute the system in vivo, providing a rich dataset of information from biochemical analysis, genetic alterations, and phenotypic changes within the tissues as well. As the first effort to simulate the complexity of joint organs, we have recently established a miniature joint system (miniJoint) composed of osteochondral, adipose, and fibrous analogs. Human bone-marrow-derived mesenchymal stem cells (hBMSCs) were differentiated into an osteochondral complex and adipose and fibrous tissues. Tissue constructs are cocultured in the miniJoint for 28 days prior to OA induction using the chemical stimulant interleukin-1β (IL-1β). Recently, we examined the genotypic and phenotypic changes in our healthy versus OA-like specimens to demonstrate the system’s ability to model healthy and OA-like knee joints. Based on these findings, we then investigated the clinical relevance of the disease model in the miniJoint using current DMOADs in clinical trials and compared our work to the reported clinical outcomes [9].

In our prior work using the miniJoint, all compounds that we examined were previously tested in either animal models or human studies [9]. An important application of MPSs is examining novel drugs’ safety and efficacy [4,7]. As a first step in exploring the utility of the miniJoint in developing DMOADs, we will test the potential of new treatment methods that have not been explored. Specifically, in OA knee joints, inflammation and cartilage degradation are often observed. In previous clinical studies, treatments targeting either one failed to reach FDA approval [10]. However, a combination of anti-inflammation and chondroinduction has not been explored, which will be tested here using the miniJoint. Specifically, BMP-7 has increased chondrocyte proliferation, matrix formation, and cell differentiation [11,12]. The use of BMP-7 as an OA therapeutic has been studied in multiple large and small animal models [12,13]. Additionally, our lab and collaborators have been investigating the role of oligodeoxynucleotides (ODNs) in suppressing the nuclear factor kappa beta (NF-κB) genetic pathway through their ability to block nuclear factor binding to promoter regions of target genes, which has been shown to be effective in inflammation reduction [14,15]. Therefore, our goal was to test the hypothesis that the combination of BMP-7 and NF-κB ODNs (referred to as ODNs from now on) can reverse OA progression using the miniJoint.

In our current work, we generated the miniJoint from hBMSCs, created a “synovitis” model using our previously established model [9], and then administered BMP-7 to the shared synovial-like fluid and ODNs to all medium types of the miniJoint. Tissues were assessed using real-time quantitative PCR (RT-qPCR), histological and immunohistochemical analysis, and LUMINEX assays. Additionally, as the first step in determining the efficacy of this new treatment, we also examined the influence of this unique therapeutic combination on human OA chondrocyte pellets. Overall, our model demonstrates the potential to develop novel DMOADs.

## 2. Materials and Methods

### 2.1. miniJoint Chip Components

All bioreactors were designed using SolidWorks 2018 software (Dassault Systèmes SE, Vélizy-Villacoublay, France). All chambers, lids, and inserts were printed using E-Shell 450 photopolymer ink with the Vida desktop 3D Printer (EnvisionTec, Dearborn, MI). Grooves were engineered into the lids and inserts to add a silicone O-ring (McMaster-Carr, Elmhurst, IL, USA) to ensure a tight seal in the miniJoint. The insert height was 4.8 mm, and the inner diameter, which housed the engineered microtissue, was 3.5 mm. The dual-flow bioreactors used to differentiate the osteochondral tissue were created using the same methodology and materials. All 3D-printed components were sterilized via an autoclave. Dimensional alterations to 3D-printed components following sterilization were not observed.

### 2.2. Engineering Individual Tissue of the miniJoint

All miniJoint tissue components were generated by differentiating human bone-marrow-derived stem cells (hBMSCs), which were isolated from total joint arthroplasty surgical waste with IRB approval (the University of Pittsburgh and the University of Washington). Similar to the previous study [9], hBMSCs were pooled to limit donor differences. Therefore, 20 cell donors were compiled, ranging from 20 to 87 years old, and these cells were characterized using prior studies [9]. hBMSCs were plated at passage 3 (P3) and expanded in the growth medium (GM; Dulbecco’s Modified Eagle Medium (DMEM, high glucose; Gibco, Grand Island, NY, USA), 10% fetal bovine serum (FBS; Gemini Bio-Products, West Sacramento, CA, USA), 1× antibiotic/antimycotic (Gibco)) supplemented with 1 ng/mL basic fibroblast growth factor (bFGF; RayBiotech, Norcross, GA). At 80–90% confluence, hBMSCs were trypsinized (trypsin-0.25% ethylenediaminetetraacetic acid; Thermo Fisher, Waltham, MA, USA). The same steps were conducted until hBMSCs reached P5. At P5, hBMSCs were trypsinized and resuspended in 15% GelMA.

A total of 17 g of gelatin type B from bovine skin (Sigma-Aldrich, St. Louis, MO, USA) was mixed with 500 mL of distilled water at 37 °C at 100 rpm until completely dissolved. Then, 13 mL of methacrylic anhydride (Sigma-Aldrich) was added, and the GelMA solution was stored overnight at 37 °C at 150 rpm. GelMA was aliquoted into 70 mL dialysis bags (Thermo Fisher), and dialysis occurred for 5 days to remove additional MA groups; the solution was then frozen at −80 °C. The frozen form was then transferred for freeze drying in a lyophilizer for 3 days. Dried GelMA was weighed, and appropriate volumes of Hank’s Balanced Salt Solution (HBSS, GE Healthcare Life Sciences, South Logan, UT) were added for a desired concentration of 15% *w*/*v* GelMA. To reach a desired p.H. of 7.4, 1 M sodium hydroxide (NaOH, Sigma-Aldrich) was incrementally added. To decrease the risk of contamination, 1% *w*/*v* antibiotic/antimycotic was added. For photocrosslinking, 0.15% LAP was added [9,16,17]. The cell-to-GelMa volume ratio was 20 million cells/mL, as established in prior studies [9]. The cell suspension was pipetted into sterile inserts and cured in situ for 2 min with 395 nm visible light illumination.

Inserts containing hBMSC-laden GelMA were placed in a dual-flow chip with an osteogenic medium (OM; DMEM, 10% FBS, 1× antibiotic/antimycotic (Gibco), 10^−7^ M dexamethasone (Sigma-Aldrich), 0.01 M β-glycerophosphate (Sigma-Aldrich), supplemented with 100 ng/mL BMP-7 (Peprotech, Rocky Hill, NJ, USA), 50 μg/mL ascorbic acid-2-phosphate, and 10 nM Vitamin D_3_ (both from Sigma-Aldrich)) and a chondrogenic medium (CM; phenol red-free Dulbecco’s modified Eagle’s medium with 1% *v*/*v* Insulin-Transferrin-Selenium-Ethonlamine (ITS), 1× antibiotic/antimycotic, and 1nM sodium pyruvate (all supplied by Gibco), 10^−7^ M dexamethasone (Sigma-Aldrich), 40 μg/mL L-proline (Sigma-Aldrich), supplemented with 10 mg/mL Transforming growth factor-β3 (TGF-β3) (Peprotech) and 50 μg/mL ascorbic acid-2-phosphate (Sigma-Aldrich)) perfused through the top and bottom channels. The culture was maintained for 28 days to form biphasic OC tissues.

Adipose tissue (AT) was formed by culturing the inserts that contained hBMSC-laden GelMA in the adipogenic medium (AM; Minimum Essential Medium (MEM Alpha, Gibco), 0.1 μM dexamethasone, 0.45mM IBMX, 0.2 mM Indomethacin (all supplied by Sigma, St. Louis, MO, USA), 1 μg/mL ITS, 1× antibiotic/antimycotic (both provided by Gibco), and 10% FBS) under static conditions for 28 days.

To generate the synovial-like fibrous tissue (SFT), hBMSCs were expanded until 80–90% confluence was reached in T300 flasks, and GM was switched to a fibrogenic medium (FM; Advanced Dulbecco’s Modified Eagle Medium (Advanced DMEM; Gibco), 5% FBS, 1× antibiotic/antimycotic, Glutamax supplement (Gibco), supplemented with 50 μg/mL ascorbic acid-2-phosphate (Sigma-Aldrich)) for 21 days. Fibroblast-like cells were then trypsinized, resuspended in 15% *w*/*v* GelMA, and pipetted into inserts.

### 2.3. Establishing the miniJoint Chip

Matured AT, SFT, and OC microtissues were assembled into miniJoint chambers, where AM, FM, and OM flowed over the top of the corresponding microtissues for tissue phenotype maintenance. A universal medium (UM; phenol red-free Dulbecco’s modified Eagle’s medium with 1% *v*/*v* ITS, 1× antibiotic/antimycotic, and 1 nM sodium pyruvate (all supplied by Gibco), 40 μg/mL L-proline (Sigma-Aldrich), supplemented with 0.5 ng/mL TGF-β3, and 50 μg/mL ascorbic acid-2-phosphate (Sigma-Aldrich)) flowed across the bottom channel that reaches the three different tissue types, allowing for crosstalk between the tissues. The bioreactors were maintained for 28 days.

### 2.4. Drug Treatment

After 28 days of tissue coculturing, IL-1β (10 ng/mL, PeproTech) was introduced to the FM stream of three miniJoints, and L-proline, ascorbic acid-2-phosphate, and TGF-β3 were removed from the UM. After 3 days of IL-1β treatment in the FM, the group 3 miniJoint was treated with 100 ng/mL BMP-7 (Peprotech) in the UM, and the group 4 miniJoint was treated with 1 µM oligonucleotide duplex 1 (ODN; Integrated DNA Technologies, Coralville, IA, USA) in all medium streams and BMP-7 in the UM, which was left for four days.

### 2.5. Histology

OC, SFT tissues, and OA pellets were fixed in 10% neutral buffered formalin (Thermo Fisher Scientific, Waltham, MA, USA) overnight at 4 °C and then dehydrated using increasing ethanol concentrations from 30% to 100%. The dehydrated samples were then cleared in xylene (Thermo Fisher Scientific), embedded in paraffin, and sectioned at a thickness of 6 μm using a manual microtome (Model RM 2255, Leica, Buffalo Grove, IL, USA). The sections were dewaxed using Histo-Clear (National Diagnostic, Atlanta, GA, USA), rehydrated with a graded series of ethanol, and stained with Hematoxylin (Sigma-Aldrich) and with 0.5% Safranin O/0.005% fast green (Sigma-Aldrich).

AT tissues were fixed in 4% paraformaldehyde (Electron Microscopy Sciences, Hatfield, PA, USA) overnight at 4 °C, dehydrated in 10%, 20%, and 30% sucrose (Sigma-Aldrich), and embedded in frozen cryo-gel (Leica Biosystems, Buffalo Grove, IL, USA). The frozen AT samples were sectioned at a thickness of 15 μm.

### 2.6. Immunohistochemistry (IHC)

OC tissues were formalin-fixed and paraffin-embedded, and 6 μm thick sections were prepared (see above). Before staining, the slides were first incubated at 60 °C for 1 h, cleared in Histo-Clear (National Diagnostic, Atlanta, GA, USA), and sequentially rehydrated. Antigen retrieval was carried out by heating the sections bathed in IHC Antigen retrieval solution (Invitrogen, Waltham, MA, USA) at 90 °C for 20 min. Then, slides were incubated in 3% hydrogen peroxide at room temperature for 10 min to block endogenous peroxidase activity. After being blocked in 1% horse serum (PK-6200 VECTASTAIN Elite ABC HRP kit, Vector Laboratories) in PBS for 45 min, the slides were incubated with antibodies against collagen II (COL2, 1:150 dilution; MA512789, Invitrogen, Waltham, MA, USA) or matrix metalloproteinase 13 (MMP13, 1:200 dilution; ab39012, Abcam, Waltham, MA, USA) overnight at 4 °C. Mouse immunoglobulin G (IgG) isotypes or rabbit IgG (both of them were from Invitrogen, Waltham, MA, USA) was used in place of the primary antibodies as negative controls. Next, a biotinylated antimouse-rabbit IgG secondary antibody (VECTASTAIN Elite ABC HRP kit, PK-6200, Vector Laboratories, Newark, CA, USA) was used. The VECTOR NovaRED peroxidase substrate kit (SK-4800, Vector Laboratories, Newark, CA, USA) was employed for signal visualization. Afterward, the tissue sections were counter-stained with Vector Hematoxylin QS (Vector laboratories, Newark, CA, USA). Sample imaging was carried out on Olympus SZX16 (Olympus, Waltham, MA, USA).

### 2.7. Total RNA Isolation and Real-Time Quantitative Reverse Transcription PCR (qTR-PCR)

Total RNA was isolated with QIAzol Lysis Reagent (Qiagen, Germantown, MD, USA), followed by RNA extraction using an RNeasy Plus Universal Kit (Qiagen). A Nanodrop 2000c Spectrophotometer (Thermo Fisher Scientific, Waltham, MA, USA)) was used to measure total RNA concentration. Reverse transcription was performed with the SuperScript^®^ VILO^TM^ cDNA Synthesis Kit (Invitrogen, Carlsbad, CA, USA). Quantitative real-time polymerase chain reaction (qRT-PCR) was performed on a Quantstudio 5 system (Thermo Fisher Scientific, Waltham, MA, USA) using PowerUP SYBR Green PCR Master Mix (Applied Biosystems, Foster City, CA, USA) according to the manufacturer’s instruction. The relative gene expression levels were calculated using the comparative (^ΔΔ^Ct) method and normalized to the gene expression of the housekeeping gene ribosomal protein L13a (RPL13a) and then further normalized to the corresponding gene expression of the control group. The sequences of primers are summarized in Table S1.

### 2.8. Luminex Assays

Eighteen hours before collecting microtissues, all medium streams were flushed with ~30 mL of basic medium (phenol-free Dulbecco’s Modified Eagle Medium supplemented with 1% *v*/*v* Insulin-Trasferrin-Selenium-Ethanolamine (Gibco)) to remove the culture medium from the microJoint and left overnight. At the time of the tissue collection, the conditioned medium was collected and centrifuged at 12,000× *g* for 10 min before −80 °C. Luminex assays were carried out using the Bio-Plex 200 system (Bio-Rad, Hercules, CA, USA). Bio-Plex Manager 6.1 software was used for data collection and analysis.

### 2.9. Enzyme-Linked Immunosorbent Assay (ELISA)

The concentration of human crosslinked C-telopeptide of type II collagen (CTX-II) in conditioned medium was measured by ELISA following the manufacturer’s instructions (BIOMATIK, Wilmington, DE, USA).

### 2.10. Pellet Culture

OA chondrocytes were used to validate the findings from studying the miniJoint. With Institutional Review Board (IRB) approval (from the University of Pittsburgh), human OA cartilage was collected from a 69-year-old female patient who underwent total knee arthroplasty. Chondrocytes were harvested by digesting minced articular cartilage with collagenase type II (10 mg/g cartilage, Worthington Biochemical Corporation, Lakewood, NJ, USA) at 37 °C for 16 h. Dissociated chondrocytes were filtered through a cell strainer. Freshly isolated chondrocytes were plated into 150 cm^2^ tissue culture flasks at P0 cultured in GM until reaching 70–80% confluence and then trypsinized and passaged. Once confluent, P1 chondrocytes were centrifuged for 10 min at 300× *g* and cultured in GM overnight. OA chondrocyte pellets were then treated with 1 μM ODNs and 100 ng/mL BMP-7 in UM with a medium change every other day for seven days.

### 2.11. Statistics

Statistical analysis was conducted via Prism 9 (GraphPad, San Diego, CA, USA). The data from this study are presented as mean ± standard deviation, with *N* ≥ 3. For datasets with two groups, a two-tailed Student’s *t*-test with Welch’s correction was used, and values were considered statistically significant at a *p*-value of at least 0.05.

## 3. Results

### 3.1. ODN+BMP-7 Treatment Increases Expression of Chondrogenic Genes in the Cartilage of the miniJoint

**Figure 1:**
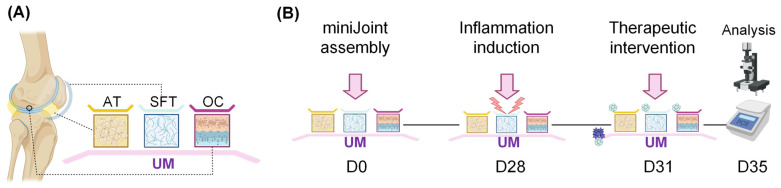
Schematic illustration of the experimental procedure. (**A**) Four tissues, including osteochondral unit (OC), adipose tissue (AT), and synovial fibroblast-like tissue (SFT), were engineered and assembled into the miniJoint. Four medium streams, demonstrated in yellow, blue, purple, and pink colors, were used to feed different tissues. Specifically, universal medium (UM) was designed to simulate synovial fluid, thus allowing crosstalk among cartilage, AT, and SFT. (**B**) After miniJoint was assembled and maintained for 28 days (D0–28), inflammation was generated by treating SFT with interleukin-1β (IL-1β). After 3 days (D31), treatments were either added to all media or only UM, mimicking “systemic” and “intraarticular” administration, respectively. Therapeutic intervention lasted for 4 days (D35), when samples were collected for data assessment.

Figure 1 summarizes the design of this study. We first generated the miniJoint and then induced synovial inflammation by introducing IL-1β into the medium that fed the SFT. A combined treatment of ODNs and BMP-7 was then applied, which were added to all media and the UM, simulating “systemic” and “local” administration, respectively. Different methods were then used to assess the therapeutic efficacy.

**Figure 2:**
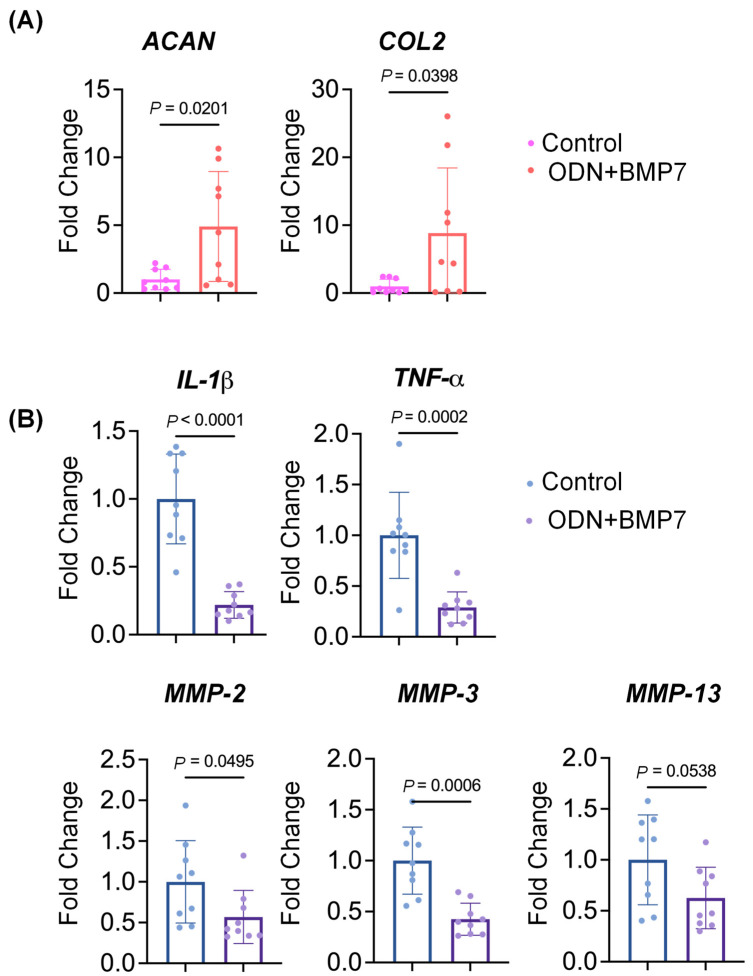
Relative gene expression levels in (**A**) cartilage and (**B**) SFT from the miniJoint treated with vehicle control (Control) or ODN+BMP-7. Data are normalized to the control group (set as 1). *N* = 9.

As shown in Figure 2A, the expression levels of representative chondrogenic genes, including aggrecan (*ACAN*) and collagen type II (*COL2*), were significantly higher in the cartilage of the miniJoint treated with ODN+BMP-7. The results obtained suggest that the combination of ODNs and BMP-7 might have a regenerative effect on OA cartilage.

### 3.2. ODN+BMP7 Treatment Decreases the Expression of Pro-Inflammatory Cytokine Genes in SFT

We then measured *IL-1β* and tumor necrosis factor-α (*TNF-α*) expression levels in SFT. As expected, both genes were downregulated with the treatment of ODN+BMP-7 (Figure 2B). Moreover, the levels of matrix metallopeptidase (*MMP*)-2, 3, and 13 were also decreased in the ODN+BMP-7 group. The decreases in inflammatory cytokines and matrix-degrading enzymes suggest that the ODN intervention was able to successfully downregulate the NF-κB pathway associated with the production of inflammatory proteins, which has also been observed in previous studies [18,19].

### 3.3. ODN+BMP7 Treatment Preserves Cartilage Integrity

**Figure 3:**
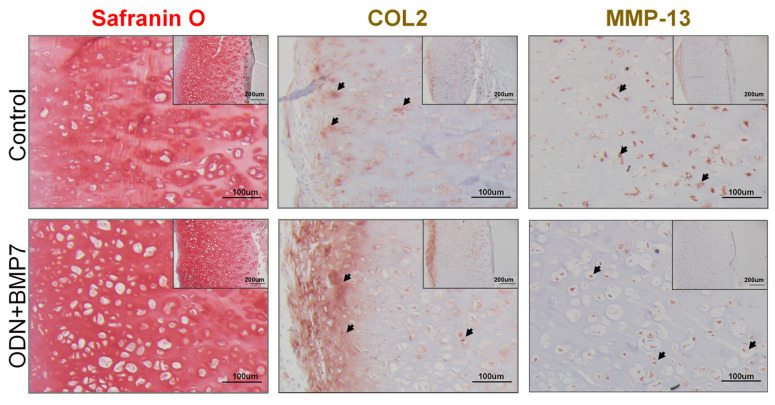
Safranin O staining and COL2 arrows pointing to COL2 positive staining and MMP-13 arrows pointing to positive MMP-13 staining IHC. Boxes in upper right hand corner are lower magnification. Bar = 200 μm. Larger boxes are higher magnification. Bar = 100 μm.

To further assess the influence of ODN+BMP-7 treatment on cartilage integrity, Safranin O staining and COL2 IHC were performed. As shown in Figure 3, cartilage tissue from the ODN+BMP-7-treated group contained more GAGs and COL2. In addition, lower MMP-13 levels were observed in the OND+BMP-7 group compared to the control group. All results implied that OND+BMP-7 preserved cartilage integrity in the inflamed miniJoint and corroborate findings from the qRT-PCR results.

### 3.4. ODN+BMP7 Reduces the Levels of Representative OA Biomarkers

**Figure 4:**
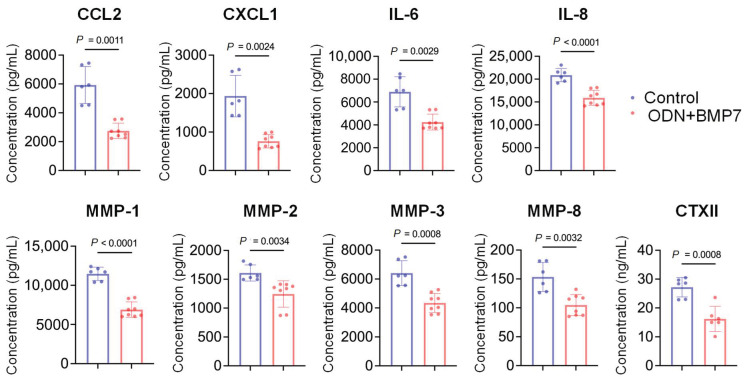
Protein levels of selected biomarkers in UM from the miniJoint treated with vehicle control or ODN+BMP-7. Monocyte chemoattractant protein-1 (CCL2), C-X-C motif chemoligand 1 (CXCL1), interleukin-6 (IL-6), interleukin-8 (IL-8), and matrix metalloproteinases (MMPs 1,2,3,8), as well as human crosslinked C-telopeptide of type II collagen (CTXII). *N* = 6.

The concentrations of selected biomarkers in the UM, which simulates the synovial fluid, were measured by Luminex or ELISA (Figure 4). Interestingly, ODN+BMP-7 treatment reduced the levels of human crosslinked C-telopeptide of type II collagen (CTXII), implying suppressed cartilage degradation. In addition, other tested pro-inflammatory cytokines and matrix metalloproteinases (MMPs) were also decreased in the ODN+BMP-7 group compared to the control group. These findings support the genetic and phenotypic data discussed previously, providing further evidence that ODN+BMP-7 treatment effectively alters cellular function in not just the synovium and cartilage tissues, but all of the tissue types of the miniJoint, as the UM is the shared medium of the system.

### 3.5. ODN+BMP-7 Promotes Chondrogenesis of Human OA Chondrocytes

**Figure 5:**
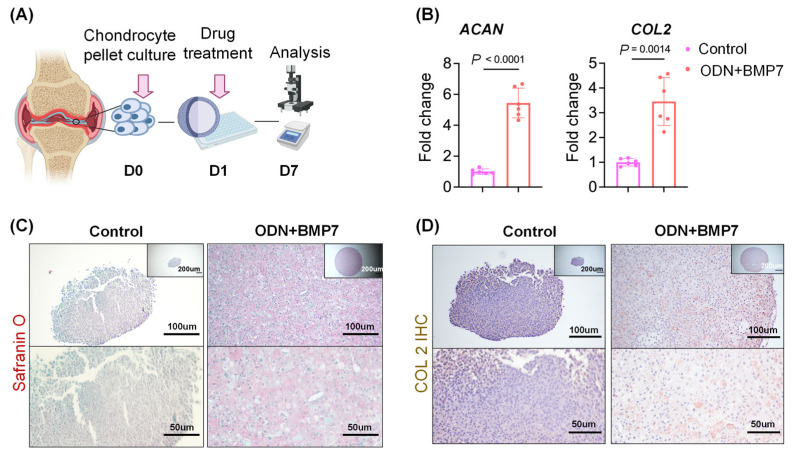
Assessing the influence of ODN+BMP-7 on human OA chondrocytes. (**A**) Schematic illustration of the study. OA chondrocytes were isolated from donors, expanded to passage 1, and then maintained in pellet culture. One day after, ODN+BMP-7 or the vehicle control was added to the culture medium. Samples were collected on day 7 and subjected to different analyses. (**B**) Relative expression levels of *ACAN* and *COL2* in pelleted chondrocytes treated with vehicle control or ODN+BMP-7. Data are normalized to the control group (set as 1). *N* = 6. (**C**) Safranin O staining of control and ODN+BMP7 treated pellets with increasing magnifications. (**D**) COL2 IHC of control and ODN+BMP7 treated pellets with increasing magnifications.

As the first step in assessing the clinical relevance of our findings on the miniJoint, OA chondrocytes were isolated, expanded, pelleted, and treated with the vehicle control or ODN+BMP-7 (Figure 5A). Similar to the findings from studying the miniJoint, ODN+BMP-7 treatment significantly increased the expression levels of *ACAN* and *COL2* (Figure 5B), which also resulted in a remarkably larger pellet size (Figure 5C,D). In addition, Safranin O staining and COL2 IHC further demonstrated that more cartilage matrix was created in the ODN+BMP-7 group compared to the control group. This indicated that OA chondrocytes provided with the treatment regained their ECM formation abilities, as seen in the miniJoint, enhancing the clinical relevance of our model.

## 4. Discussion

Organs-on-chips and microphysiological systems have been proposed as novel tools to study OA and develop treatments [6,20,21]. There have been several models using many methods for replicating one or more components of the synovial knee joint. Our lab has extensively studied the fabrication of engineered tissues constituting the knee joint. Our first study demonstrated the creation of osteochondral tissue using hBMSCs and their response to IL-1β treatment [17]. We then began investigating a similar model using induced pluripotent stem cells (iPSCs) to model OA and test therapeutics, in which we reported the successful differentiation of iPSCs into the osteochondral tissue, induced OA using IL-1β, and tested the effect of Celecoxib for OA treatment [22]. The work with hBMSCs allowed for the further engineering of adipose and fibrous tissue analogs to develop the complete miniJoint demonstrated in this study [9]. For example, using the miniJoint, we demonstrated the critical role of synovial tissue in escalating inflammation and promoting cartilage degradation. From our system, we can assess phenotypic alterations using histology and immunohistochemistry and genotypic changes and analyze protein output through LUMINEX arrays, providing a plethora of information. In a preliminary study that tested around 10 compounds, we individually defined the potential of BMP-7 and NF-κB ODNs. The miniJoint tested in this study is composed of crosslinkable resin allowing for the precise 3D printing of all components [9,22]. The plug-and-play features of our system allow for the continued analysis of tissue–tissue interactions during OA.

Further investigations of organ-on-chip systems have been conducted. Specifically, a cartilage-on-chip model was developed by Occhetta et al. to investigate the role of mechanical loading on OA pathogenesis and test therapeutics [23]. Dwivedi et al. fabricated an MPS from osteochondral tissue plugs and synovial capsule explants and used a mechanical loading device to induce post-traumatic osteoarthritis (PTOA) [24]. They assessed the initiation of PTOA in their model to observe early inflammatory markers produced by the synovium that led to further cartilage degradation [24]. Mondadori et al. investigated monocyte extravasation using microfluidics [25]. They were the first to report the creation of an extravasation model for the knee joint, as other extravasation organ-on-chips have been used to investigate cancer cell and neutrophil behavior. Other work has focused on creating the different cartilage zones in a nutrient-gradient-based chip using primary equine cells. Here, Rosser et al. created a 1:1 articular cartilage model through the redifferentiation of chondrocytes in a tissue-engineered cartilage hydrogel [26]. Organ-on-chip models provide a platform to model specific disease states and allow for unique tissue analysis. One such example comes from Rothbauer and colleagues, who used label-free non-invasive optical light to study alterations to cartilage organoids for Rheumatoid Arthritis [23,27].

Bone morphogenic protein-7 (BMP-7), also known as osteogenic protein-1, has been investigated in a variety of in vitro studies as an OA therapeutic and cartilage extracellular matrix (ECM) regenerative factor [28,29,30]. BMP-7 treatment successfully upregulated chondrogenic ECM proteins during OA, such as collagen type II, aggrecan [31], decorin, fibronectin, and hyaluronic acid [32]. An essential aspect of these BMP-7 studies is the maintenance of the cartilage phenotype, and more recent studies have begun to investigate the ability of BMP-7 to suppress chondrocyte hypertrophy [33]. BMP-7 has been clinically approved for both bone and cartilage regenerative purposes [33] and is currently in clinical trials as an OA therapeutic [34].

Nuclear factor-κB is a well-known transcription factor associated with chondrocyte development; NF-κB plays an important role in the progression of OA via the production of inflammatory cytokines and chemokines such as MMP-13 and ADAMTS5 [35]. During OA, chondrocytes demonstrate increased inflammatory and hypertrophic markers, which are thought to be linked to NF-κB signaling [36]. Multiple studies have assessed the efficacy of blocking NF-κB binding via the use of inhibitors of NF-κB (IκB) proteins, which bind to NF-κB in the cytoplasm and cause the activation of IκB kinases (IKKs), allowing for the phosphorylation of IκBs in the cell cytoplasm [37,38]. Another study used short hairpin RNAs through retroviral transduction in human chondrocyte micromass culture and found that the ablation of IKKα caused cells to increase collagen type II production and have increased proliferation compared to cultures with IKKβ knockdown [38]. Furthermore, targeting the NF-κB axis has shown promising results in suppressing catabolic enzymes in vitro and in vivo [39]. Based on these findings, we further investigated the role of potential NF-κB-suppressing factors, oligodeoxynucleotides (ODNs).

A new NF-κB therapeutic, ODNs, is being investigated for its role in disrupting NF-κB binding to promoter regions, thus interfering with NF-κB binding [18]. This anti-inflammatory therapeutic is being heavily studied by members of our team for the treatment of periprosthetic osteolysis by total joint arthroplasty wear particles, which cause inflammation and the subsequent activation of the NF-κB pathway. They use in vivo murine models to study ODN efficacy as an anti-inflammatory intervention in young and old mice, which is essential for OA, as many individuals with OA are older than 65 years [19]. Their studies reported the ability of ODNs to bind to the NF-κB p65:IKb complex promoter region, suppressing the genetic activation of key inflammatory genes [15]. One in vivo study used polyethylene wear particles to induce chronic inflammation for 6 weeks and employed ODNs in the last 3 weeks. The results demonstrated that the ODNs suppressed inflammation [15]. More recently, members of our team conducted a similar study in aged mice to determine the effects of femoral infusion of ODNs in an aged osteolysis model [15]. ODNs were able to alter the macrophage phenotype from M1 to an M2 anti-inflammatory phenotype in young and aged mice. However, NF-κB ODNs were less effective in older mice, which was thought to be linked to the chronic inflammatory state already seen in aged mice and the limited efficacy of NF-κB caused by aging [15]. Despite the results in aged mice, it was determined that ODNs were effective and safe in the mouse model and could be a novel therapeutic for targeting the NF-κB pathway in OA [15,19].

Given their respective chondrogenic and anti-inflammation functions, we examined a combination of BMP-7 and ODNs as a potential therapeutic for OA. Based on our data, we determined that there was an increase in aggrecan and collagen type II in the BMP-7/ODN treatment group compared to the IL-1β-treatment-only group.

While our system presents many advantages, our model is limited in the cost of maintaining the tissues in culture for 28 days and the necessary parts for tissue chip construction. Additionally, our model replicates OA as a synovitis-on-chip method, as OA is induced in the miniJoint through the introduction of IL-1β into the synovial-like fibrous tissue at a concentration of 10ng/mL. This concentration of IL-1β is not consistent with that seen in the native pathology of OA, in which concentrations of IL-1β are much lower, around 20–30 pg/mL [4]. Although our previous study showed that cartilage in the IL-1β-treated miniJoint displayed reduced *COL2* and *ACAN* and increased *MMP-1* and *MMP-13* expression [9], more physiologically relevant OA-inducing methods such as mechanical loading should be investigated to model OA in the miniJoint and test the efficacy of ODN+BMP-7. Second, the synovial-like fibrous tissue also lacks immune cells, which contribute significant amounts of inflammatory factors to the OA disease state [40,41]; incorporating macrophages or immune cells into our tissue chip would provide further insight into how synovitis contributes to OA progression. Third, the doses of these two compounds used in this study were selected based on previous publications. Fourth, we did not explore the potential influence of the BMP-7/ODN ratio on reducing the OA phenotype. Lastly, primary human OA chondrocytes from surgical waste were used to validate the effects of BMP-7 and ODNs, and only one donor was obtained for this study. To support the clinical relevance of the miniJoint, it is essential to obtain results from multiple OA donors. This combination of treatments has yet to be tested in animals or the clinical setting. Further analysis in vivo should be conducted to better understand the joint implications of the combined therapy for knee OA.

## Data Availability

All data supporting reported results have been included.

## Supplementary Materials

The following supporting information can be downloaded at https://www.mdpi.com/article/10.3390/biom13020384/s1. Table S1: Sequences of primers that were used in this study.
