# Supplementary material for: Using Microphysiological System for the Development of Treatments for Joint Inflammation and Associated Cartilage Loss—A Pilot Study
Source: Biomolecules. 2023 Feb 17;13(2):384. doi: 10.3390/biom13020384 (PMC9952916; doi:10.3390/biom13020384)
Supplement: Supplementary file 1 [file biomolecules-13-00384-s001.zip › biomolecules-2202101-supplementary.pdf]

Table S1: Sequences of primers that were used in this study.

Gene	Forward primer (5′->3′)	Reverse primer (5′->3′)
RPL13a	GCCATCGTGGCTAAACAGGTA	GTTGGTGTTCATCCGCTTGC
COL2	GGATGGCTGCACGAAACATACCGG	CAAGAAGCAGACCGGCCCTATG
ACAN	AGTCACACCTGAGCAGCATC	AGTTCTCAAATTGCATGGGGTGTC
MMP-2	TACAGGATCATTGGCTACACACC	GGTCACATCGCTCCAGACT
MMP-3	CGGTTCCGCCTGTCTCAAG	CGCCAAAAGTGCCTGTCTT
MMP-13	ACTGAGAGGCTCCGAGAAATG	GAACCCCGCATCTTGGCTT
IL-1B	ATGATGGCTTATTACAGTGGCAA	GTCGGAGATTCGTAGCTGGA
TNF-a	CCTCTCTCTAATCAGCCCTCT	GAGGACCTGGGAGTAGATGAG
